# Pectin from *Citrus unshiu* Marc. Alleviates Glucose and Lipid Metabolism by Regulating the Gut Microbiota and Metabolites

**DOI:** 10.3390/foods12224094

**Published:** 2023-11-11

**Authors:** Yanming Ren, Shuifang Mao, Yujun Zeng, Shiguo Chen, Jinhu Tian, Xingqian Ye

**Affiliations:** 1 College of Biosystems Engineering and Food Science, Zhejiang University, Hangzhou 310058, China; yanmingren@yeah.net (Y.R.); msf15261598502@163.com (S.M.); 22213036@zju.edu.cn (Y.Z.); chenshiguo210@163.com (S.C.); jinhutian@126.com (J.T.); 2 Zhongyuan Institute, Zhejiang University, Zhengzhou 450000, China

**Keywords:** pectin, glycolipid metabolism, gut microbiota

## Abstract

The effects of pectin from *Citrus unshiu* Marc. on glycolipid metabolism, the morphologies of the pancreas and epididymal fat, the gut microbiota, and the metabolites of short-chain fatty acids (SCFAs) in db/db mice were investigated in this study. The results indicated that pectin reduced the levels of fasting blood glucose, glycated serum protein, triglycerides, total cholesterol, and low-density lipoprotein cholesterol while increasing the levels of high-density lipoprotein cholesterol. Meanwhile, pectin could improve the morphology of islet cells and inhibit the hypertrophy of adipocytes. Additionally, pectin not only regulated the intestinal flora dysbiosis in db/db mice, as shown by the increasing proportion of *Firmicutes*/*Bacteroidetes* and the relative abundance of *Ligilactobacillus*, *Lactobacillus*, and *Limosilactobacillus*, but also remedied the metabolic disorder of SCFAs in db/db mice. These results suggest that pectin could promote glucose and lipid metabolism by regulating the intestinal flora with changes in SCFA profile. This study proves that pectin might serve as a new prebiotic agent to prevent the disorder of glycolipid metabolism.

## 1. Introduction

Type II diabetes mellitus (T2DM) is known as a chronic metabolic disease featured by hyperglycemia, hyperlipidemia, and insulin resistance (IR) induced by insufficient insulin secretion or insulin deficiency [1,2,3]. Recently, the incidence of T2DM has been increasing all over the world, and the cost of treating T2DM and its complications has brought a huge burden to the world’s economy and health system. The treatment of diabetes involves many aspects, including nutritional therapy, insulin therapy, antidiabetic drug therapy, health education, etc. [4]. Currently, synthetic diabetes drugs mainly include thiazolidinediones, biguanides, sulfonylureas, α-glucosidase inhibitors, GLP-1 analogs, etc. However, hypoglycemic drugs cause serious side effects, such as hypoglycemia, weight gain, gastric intestinal side effects, edema, etc. [5,6,7]. Therefore, it is vital for the prevention and remedy of T2DM to use safe and effective natural hypoglycemic functional foods.

Plant polysaccharides are natural bioactive substances that exist in plant tissues. Many natural plant polysaccharides have been proven to possess good hypoglycemic and hypolipidemic activities and have become one of the research focuses in the field of pharmaceutical and functional foods [8,9,10,11,12]. Many studies have proven that polysaccharides can improve T2DM, including by improving islet cell function, increasing satiety, reducing glucose diffusion, and modulating the gut microbiota [13,14,15,16,17]. Pectin is a kind of acidic heteropolysaccharide widely presented in the primary cell wall and cell middle lamella of higher plants and exhibits a great deal of biological activities, including anti-inflammatory and hypoglycemic activities, inhibiting colon cancer, reducing atherosclerosis, preventing colitis, etc. [18,19,20,21,22]. Pectin is normally composed of three structural domains: homogalacturonan (HG), type I rhamnogalacturonan-I (RG-I), and type II rhamnogalacturonan (RG-II) [18]. RG-I pectin is one of the main structural domains of pectic polysaccharides, accounting for about 20–35%. It is widely found in fruits and vegetables and has been proven to show numerous biological activities, including anti-diabetic and anti-inflammatory activities, immunity enhancement, intestinal prebiotics, etc. [18,21]. In addition, dietary fiber can regulate intestinal health, control blood glucose, reduce blood lipids, and relieve inflammation by regulating the intestinal flora and its metabolites [2]. Jiao et al. proved that oral administration of 100 mg/kg ginseng RG-I polysaccharides could effectively reduce blood glucose and increase insulin levels and hepatic glycogen production in T2DM rats [23]. In terms of improving hyperlipidemia, RG-I also reduces blood lipid levels by reducing liver fat accumulation, improving lipid metabolism, and promoting butyrate production and white fat browning [24]. Therefore, pectic polysaccharides exhibit good hypoglycemic and hypolipidemic activities.

During the processing of canned citrus, a large amount of acid and basic processing water will be produced, which is rich in pectin and flavonoids [25]. Their direct discharge will not only produce a lot of pollution but also cause the waste of active ingredients. Previous studies have proven that the RG-I structure of pectin extracted from citrus alkali water has a higher proportion of RG-I and possesses good biological activity [24]. Zhu et al. pointed out that RG-I pectin recovered from citrus processing water could prevent obesity by reversing the imbalance of intestinal flora induced by a high-fat diet and promoting the browning of white fat, and its effect was better than that of HG pectin. They speculate that the monosaccharide composition may be the key structural property related to the polysaccharide–microbiome regulating effect, and RG-I pectin abundant in neutral sugars is more easily fermented than HG pectin abundant in acidic sugars [24].

At present, few studies have focused on the hypoglycemic activity of pectin, especially on the hypoglycemic and hypolipidemic effects of different types of pectin in vivo. Therefore, the aim of the present study was to explore the effects of different types of pectin collected from citrus canning processing water on glucose and lipid metabolism in db/db mice. This study has proven that pectin might serve as a new prebiotic agent to prevent the disorder of glycolipid metabolism.

## 2. Materials and Methods

### 2.1. Materials

Pectin from acid water (PA) and basic water (PB) was recovered from citrus canning processing water in our previous study [25]. The commercial pectin (CP) (CAS: 9000-69-5) was bought from Sigma-Aldrich (Shanghai, China). PA is mainly composed of rhamnose (3.47%, mol %), arabinose (25.78%, mol %), galactose (8.89%, mol %), and galacturonic acid (55.87%, mol %) with an average molecular weight of 537.7 kDa. PB is mainly composed of rhamnose (6.19%, mol %), arabinose (40.38%, mol %), galactose (13.71%, mol %), and galacturonic acid (32.61%, mol %) with an average molecular weight of 656.5 kDa. CP is mainly composed of rhamnose (5.83%, mol %), arabinose (2.22%, mol %), galactose (9.83%, mol %), and galacturonic acid (74.48%, mol %) with an average molecular weight of 405.6 kDa.

### 2.2. Animals and Treatment

Thirty male C57BL/KsJ-db/db mice and six db/m mice (6–8 weeks old) were bought from Cavens Animal Co., Ltd. (Changzhou, China) (SCXK (Su) 2016-0010), housed under defined environment conditions (12 h light/dark cycle, relative humidity of 45–50%, and room temperature of 25 ± 2 °C). The mice were allowed free access to food and water. The animal experiments (No. ZJCLA-IACUC-20020069) were approved by the Experimental Animal Welfare Ethics Committee of Zhejiang Experimental Animal Center and carried out according to the Guide for the Care and Use of Laboratory Animals.

After one week of adaptation, the experimental mice were randomly grouped into five groups: the T2DM model control group (MC), the metformin-positive control group (PC, 100 mg/kg), the pectin from acid water group (PA, 200 mg/kg), the pectin from basic water group (PB, 200 mg/kg), the commercial pectin group (CP, 200 mg/kg), and db/m mice as the control group (NC). Each group was fed with a standard chow diet. The oral administration of pectin was carried out by gavage for 4 weeks, and the mice of the NC and MC groups were gavaged with the same amount of ultrapure water every day. The body weight and blood glucose levels of the mice were determined and recorded every week. On the last day of administration, all mice were fasted for 12 h and anesthetized, and blood samples were collected after their eyes were removed. The serum, pancreas, colon, and epididymal fat were collected and stored at −80 °C for further experiment.

### 2.3. Measurement of Fasting Blood Glucose and Glycated Serum Protein

The blood samples underwent a meticulous centrifugation process, executed at 3000 revolutions per minute, under a refrigerated setting of 4 °C, for a duration of 15 min, resulting in the extraction of serum. The fasting blood glucose (FBG) levels of the mice were subjected to weekly assessments. The FBG levels of the murine subjects were ascertained by means of Accu-Chek Performa (courtesy of Roche Diagnostics, headquartered in Mannheim, Germany). These FBG assessments were conducted by prudently collecting blood from the mice’s tail veins after a mandatory overnight fasting.

Simultaneously, the concentrations of glycated serum protein (GSP) within the serum samples were diligently determined. This assay was conducted through the application of commercial reagent kits, acquired from the renowned Nanjing Jiancheng Bioengineering Institute, which operates from the vibrant city of Nanjing, China.

### 2.4. Serum Lipids Function in Mice

The lipid profiles within each experimental group, encompassing triglycerides (TG), total cholesterol (TC), low-density lipoprotein cholesterol (LDL-C), and high-density lipoprotein cholesterol (HDL-C), were meticulously assessed by employing commercially available kits sourced from the Nanjing Jiancheng Bioengineering Institute, situated in the vibrant city of Nanjing, China.

### 2.5. Hematoxylin and Eosin Analysis

The freshly obtained pancreatic, epididymal fat, and colon tissue samples were meticulously preserved in a 4% paraformaldehyde solution at ambient room temperature. Subsequently, the tissues underwent thorough immersion in absolute ethyl alcohol for 24 h before being meticulously embedded in paraffin wax. The resulting tissue blocks were then meticulously sectioned into slices, each of which was a mere 5 μm in thickness. Following this, the tissue sections were subjected to hematoxylin and eosin (H&E) staining, allowing for detailed histological examination. The images of the pancreatic, epididymal fat, and colon tissue were then meticulously observed and captured under the illumination of a light microscope.

### 2.6. Change of Gut Microbiota

Gut microbiota gene sequencing from the fecal samples embarked on a comprehensive process. It commenced with the application of DNA extraction kits for the meticulous extraction of genetic material from the fecal samples. The quality of the extracted DNA was evaluated through 0.8% agarose gel electrophoresis, while quantification was ascertained via the meticulous utilization of a UV spectrophotometer.

Subsequently, the 16S rDNA hypervariable region (V3-V4) was amplified through PCR, utilizing DNA as the fundamental template. The primers deployed for this purpose were ACTCCTACGGGAGGCAGCA and GGACTACHVGGG TWTCTAAT. Following PCR amplification, the products underwent scrutiny through 2% agarose gel electrophoresis and were subsequently subjected to purification using an AXYGEN gel extraction kit.

Following the initial quantification results obtained from electrophoresis, a Quant-iT PicoGreen dsDNA detection kit was harnessed for the fluorescent detection of the PCR amplification products. In light of the quantification outcomes, the samples were judiciously blended in their respective proportions. Building on Illumina MiSeq sequencing technology, the preparation of sequencing libraries was accomplished using Illumina’s TruSeq Nano DNA LT Library Prep Kit (Shanghai, China).

Sequencing entailed the performance of 2300 bp paired-end sequencing via the MiSeq sequencer, accompanied by the application of the corresponding MiSeq reagent Kit V3 (600 cycles). The entire analytical framework was rooted in the foundation of sequencing reads and operational taxonomic units (OTUs).

### 2.7. Determination of SCFAs

The SCFA content of the fecal samples was determined by GC according to the method of the previous study with minor modifications [26]. In brief, the fecal samples were initially weighed with precision in test tubes, followed by the addition of distilled water at a ratio of 1:6 (feces to distilled water). The mixture was then homogenized for 30 s using a handheld homogenizer, subjected to 30 min of sonication, and subsequently centrifuged at 13,000 rpm for 10 min at 4 °C. The resulting supernatant was filtered through a syringe filter with a 0.22 μm pore size. Gas chromatography (performed on an Agilent Technologies instrument, Stockport, UK) utilized a 30 m × 0.25 mm × 0.25 μm HP-INNOWax column (No. 19091N-133; Agilent Technologies, Santa Clara, CA, USA) in conjunction with a flame ionization detector (Agilent Technologies). The quantification of short-chain fatty acids (SCFAs) was accomplished by the comparing peak areas to those of established chemical standards.

### 2.8. Statistical Analysis

Each experiment was conducted in triplicate, and the results are presented as means with accompanying standard deviations. Statistical analysis was performed using analysis of variance (ANOVA) via Duncan’s test, employing SPSS software version 21.0 (IBM software, Chicago, IL, USA). The significance threshold was established at *p* < 0.05.

## 3. Results

### 3.1. Pectin Reduced Body Weight in db/db Mice

**Figure 1:**
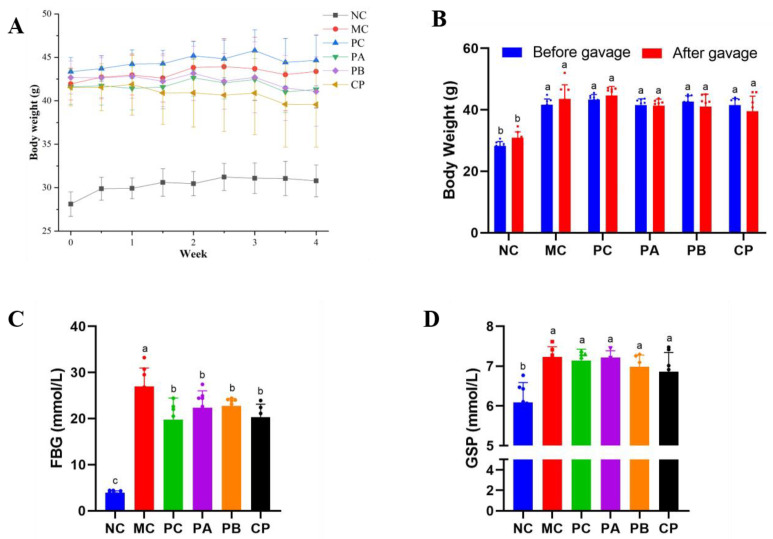
Effect of different types of pectin on body weight (**A**,**B**), FBG (**C**), and GSP (**D**) in db/db mice (*n* = 6). Significant differences were assessed by one-way ANOVA. Different letters over the bars indicate significant differences (*p* < 0.05).

The body weight changes of the mice in each group are shown in Figure 1A,B. Compared with the NC group, the weight of the mice in the experimental group was more than 10 g, indicating that the db/db mice were obese mice [5,10]. The weight of the NC group increased steadily during the feeding process, but no significant differences were observed before and after gavage (*p* > 0.05). The MC and PC groups also increased slightly, while the weight of the mice in the different pectin gavage groups decreased slightly without any significant difference.

### 3.2. Pectin Decreased FBG and GSP in db/db Mice

Figure 1C,D shows the FBG and GSP levels of the mice in each group. Compared with the NC group, the FBG in the MC group was significantly increased (*p* < 0.05), indicating that the model was built successfully. After pectin gavage, FBG improved significantly, but no significant differences among the different groups (PC, PA, PB, and CP groups) were observed. GSP can reflect the control level of blood glucose in the body for about three weeks. Compared with the NC group, the level of GSP in the MC group was significantly increased while it decreased after gavage with pectin. These results indicate that pectin with different structures could improve FBG to some extent.

### 3.3. Pectin Improved Lipid Profiles in the db/db Mice

**Figure 2:**
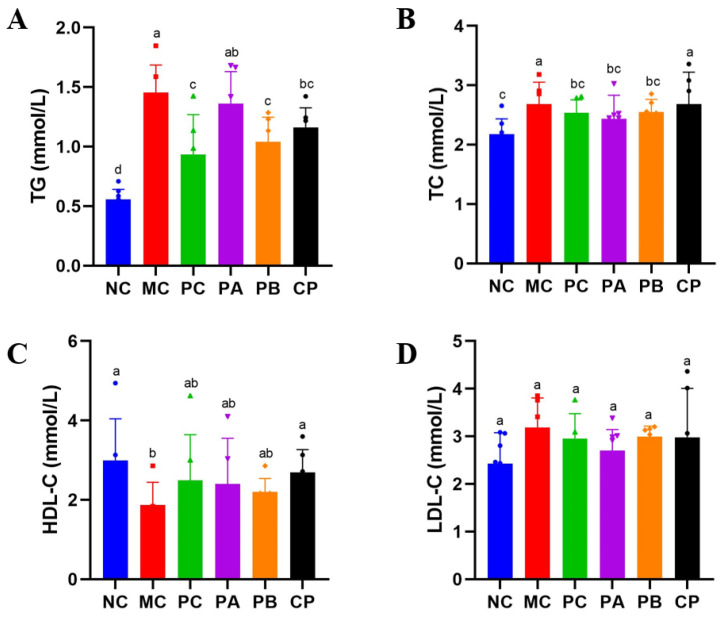
Effect of different types of pectin on serum biochemical indicators in the db/db mice. (**A**) TG, (**B**) TC, (**C**) HDL-c, and (**D**) LDL-c (*n* = 6). Significant differences were assessed by one-way ANOVA. Different letters over the bars indicate significant differences (*p* < 0.05).

Figure 2 shows the results of serum lipids in the db/db mice. Compared with the NC group, the TG and TC of the MC mice significantly increased (*p* < 0.05), while HDL-C decreased. However, pectin could reduce TG and TC and increase HDL-C in the db/db mice after gavage for 4 weeks, and there was no significant difference in the levels of LDL-C among the different groups (*p* > 0.05).

### 3.4. Pectin Ameliorated the Morphology of the Pancreas, Epididymal Fat, and Colon Tissue

**Figure 3:**
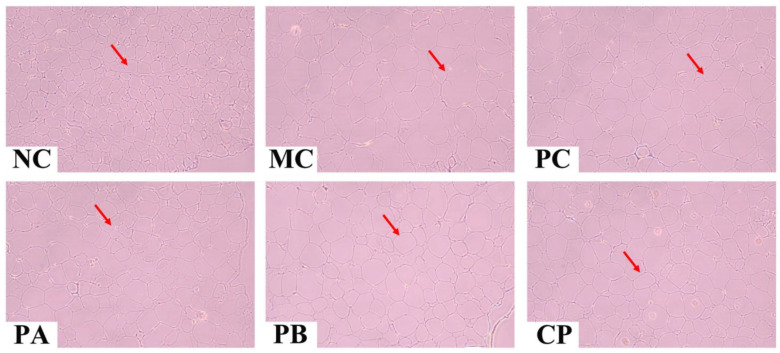
Effect of different types of pectin on epididymal fat histomorphology in the db/db mice (*n* = 6). The red arrow marks the location of the adipocyte distribution.

**Figure 4:**
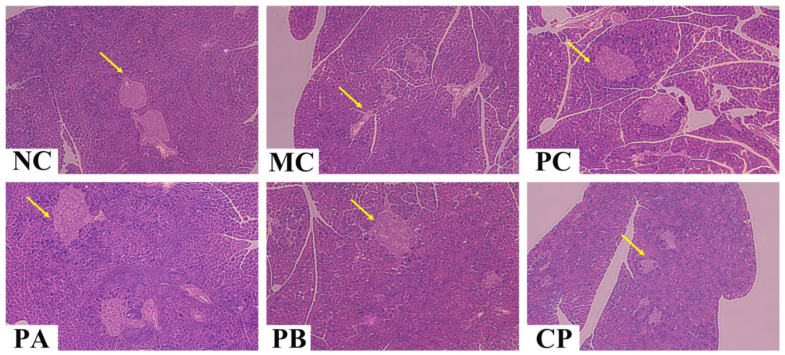
Effect of different types of pectin on pancreas histomorphology in the db/db mice (*n* = 6). The yellow arrow marks the location of the islet cell distribution.

**Figure 5:**
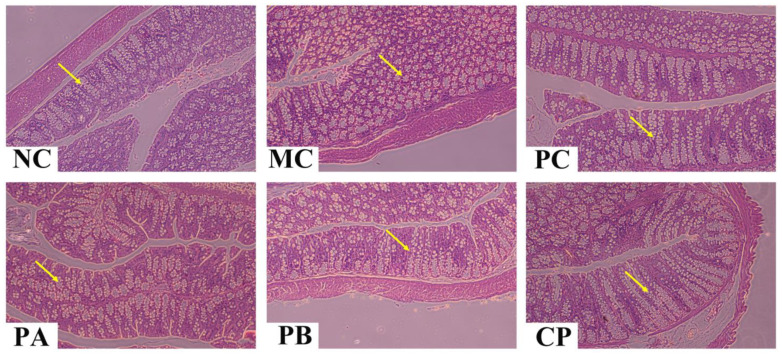
Effect of different types of pectin on colon histomorphology in the db/db mice (*n* = 6). The yellow arrows mark the location of the goblet cell distribution.

Figure 3, Figure 4 and Figure 5 show the images of the tissue (epididymal fat, pancreas, and colon) sections of the mice in each group. Compared with the NC group, the adipocytes in the model group were significantly larger, with excessive hypertrophy and abnormal hyperplasia, but after gavage with different pectins (PA, PB, and CP), the trend of adipocyte volume increase in the model group was reversed, indicating that pectin could inhibit the hypertrophy of adipocytes by alleviating the disorder of lipid metabolism in db/db mice. For the morphologies of the pancreatic tissue, compared with the NC group, the pancreas of the MC mice was severely damaged, the islet cells were destroyed, and the structure was incomplete. When treated with different pectins (PA, PB, and CP), the morphology of islet cells in each group was improved and the tissue was repaired partially. Regarding colon tissue, the MC mice showed fewer colonic crypts and slightly fewer goblet cells compared to the NC group. After pectin gavage (PA, PB, and CP), the number of crypts increased, the crypts became deeper, and the number of goblet cells was higher than that in the MC group.

### 3.5. Pectin Modulated the Gut Microbiota in the db/db Mice

**Figure 6:**
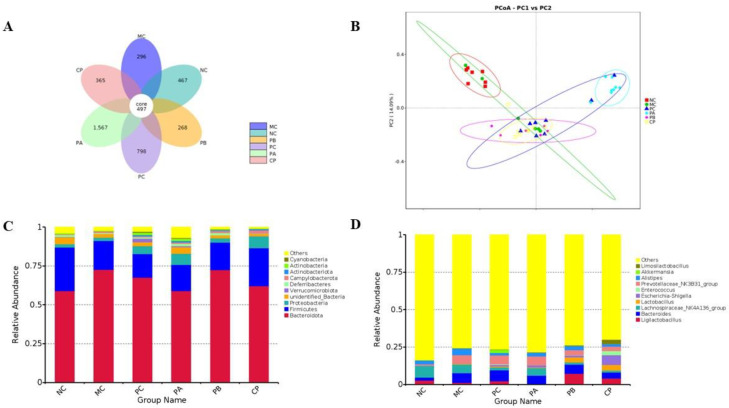
Effect of different types of pectin on the overall structure of the gut microbiota in the db/db mice (*n* = 6). (**A**) Venn diagram of OTUs, (**B**) principal coordinate analysis (PCoA), and (**C**,**D**) relative abundance at the phylum and genus levels.

The effects of the different pectins on the gut microbiota characteristics of the db/db mice were studied by 16S rDNA sequencing. The Venn diagram analysis of OTUs indicated that all groups shared 497 OTUs (Figure 6). The unique OTUs for the NC, MC, PC, PA, PB, and CP groups were 467, 296, 798, 1567, 268, and 365, respectively. Principal coordinates analysis (PCoA) was used to divide the samples into cohesive groups according to different distance thresholds and to identify the similarity of the gut microbiota among the different groups. PCoA analysis showed that the NC group was mainly in the first quadrant, and the other groups were separated from the NC group; the rest of the MC group was also separated after supplementing different structural pectins, showing that different pectins affect the gut microbiota composition of db/db mice. These results suggested important changes in the gut microbial profile of the db/db mice after pectin supplementation.

**Figure 7:**
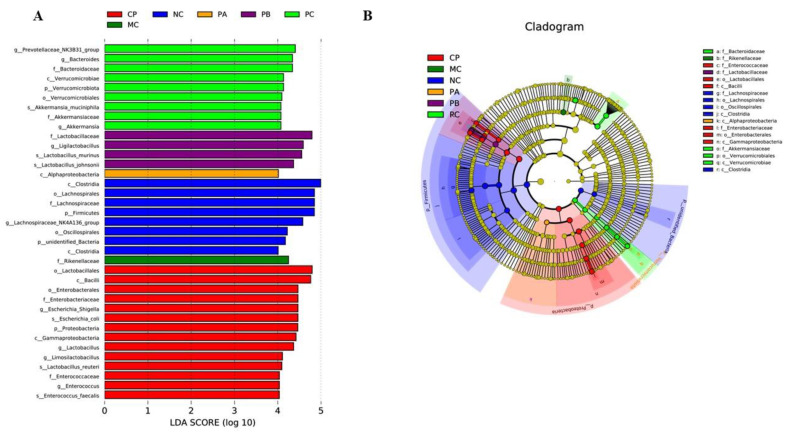
Identification of the characteristic gut microbiota in different groups by LEfSe analysis (*n* = 6). (**A**) Characteristic microbial taxa among groups meeting an LDA significance threshold > 4. (**B**) Multilevel species hierarchy of the LEfSe analysis.

In order to further analyze the effects of different pectins on the gut microbes of the db/db mice, the relative abundance compositions of each group of gut microbiota at the phylum and genus levels were analyzed according to the OTU annotation results. At the phylum level, *Bacteroidetes* and *Firmicutes* accounted for the largest proportion of phyla, which was consistent with previous studies [27]. Compared with the NC group, the F/B (*Firmicutes*/*Bacteroidetes*) ratio of the MC group decreased, and the F/B value increased after gavage with different pectins, especially in the CP group. At the genus level, compared with the NC group, the MC group had a higher relative abundance of *Bacteroides*, *Prevotella,* and *Alistipes*. Compared with the MC group, the PB and CP groups significantly increased the relative abundance of *Ligilactobacillus*, *Lactobacillus,* and *Limosilactobacillus*. Meanwhile, the relative abundance of *Akkermansia* in the PC group increased significantly. Linear discriminant analysis effect size (LEfSe) analysis serves as a powerful tool for unearthing and elucidating discriminative markers within data of elevated complexity, effectively pinpointing the characteristics that offer the most compelling insights into the distinctions among species and the repercussions of these very traits. Upon subjecting the gut microbiota to LEfSe analysis, a rich tapestry of 37 distinct species emerged as pivotal contributors to the relative abundance variations across the various experimental groups. Notably, within this mosaic of microbial diversity, the NC group could be attributed to the prominence of twelve bacterial species, while the MC group made its mark with one. Similarly, the PA group’s footprint was discernible through a singular bacterial entity, whereas the PB group left its imprint with four bacterial species. The CP group, on the other hand, exhibited its distinctiveness through a generous assembly of 14 bacterial species (Figure 7). Among them, the main differential bacterium in the PB group was *Lactobacillaceae*, and the main differential bacteria in the CP group were *Lactobacillalles* and *Bacilli*.

### 3.6. Pectin Promoted the Generation of SCFAs in db/db Mice

**Figure 8:**
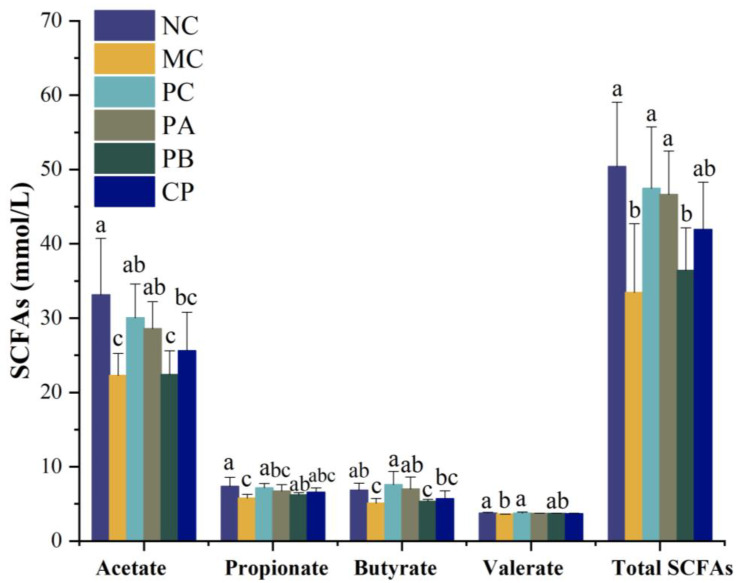
Effect of different types of pectin on the changes in short-chain fatty acids in the db/db mice (*n* = 6). Significant differences were assessed by one-way ANOVA. Different letters over the bars indicate significant differences (*p* < 0.05).

Figure 8 shows the levels of SCFAs in the feces of mice in each group. Compared with the NC group, the content of total SCFAs in the feces of the mice in the MC group was significantly decreased, and the level of total SCFAs was significantly increased when pectin was administered by gavage. Especially for the PA group, which may be related to its structural composition. In addition, the concentrations of acetic acid, propionic acid, and butyric acid also increased significantly with pectin (PA, and CP) gavage. Therefore, pectin can promote the generation of SCFAs in db/db mice.

## 4. Discussion

Type 2 diabetes mellitus (T2DM) stands as a persistent ailment distinguished by the presence of elevated blood glucose levels, often stemming from a shortage in insulin secretion or complications related to pancreatic function. The fundamental mechanisms underpinning its pathogenesis encompass the intricate interplay of insulin resistance (IR) and disruptions in the regulation of both glucose and lipid metabolism [28]. Of these mechanisms, IR assumes a pivotal role, not merely as the instigating force behind T2DM’s pathogenic progression but as the primary influencer of the myriad of diabetic complications that accompany it, including the enduring elevation of blood glucose levels, aberrations in lipid metabolism, diabetic nephropathy, and vascular diseases [29,30]. Consequently, one promising avenue for managing T2DM is the amelioration of IR and the restoration of equilibrium in glucose and lipid metabolism.

Plant polysaccharides refer to macromolecular substances that link multiple monosaccharides of the same or different types through α or β-glycosidic bonds and are widely found in plants [31]. A variety of plant polysaccharides from different sources can effectively improve T2DM and delay the symptoms related to the onset of T2DM [32]. In this experiment, db/db mice were used as model animals to study the effects of different pectins on glucose and lipid metabolism in vivo. After 4 weeks of birth, the db/db mice gradually developed the characteristics of obesity, hyperglycemia, hyperlipidemia, and IR. After 8–12 weeks, their blood glucose continued to rise until chronic complications and pancreatic β cell failure occurred. The mice died within about one month. The pathogenesis of db/db mice is very similar to that of human T2DM, and it is an ideal animal model for the study of human T2DM.

Before the experiment, the blood glucose level of the db/db mice was high, and there were symptoms such as obesity, polydipsia, and polyuria. The weight of the mice decreased after gavage with different types of pectin, but no significant differences among them were observed. FBG was significantly elevated in the MC group compared to the NC group, indicating that the model was built successfully. Additionally, after 4 weeks of pectin gavage treatment, the FBG of the three treatment groups was lower than that of the MC group, indicating that pectin had some hypoglycemic activity. This is also consistent with previous related reports [23]. There was no significant difference in the level of reduced FBG of the three pectin species, and the reason may be that their monosaccharide composition was the same with only different proportions. In addition, the onset of T2DM is closely related to the body’s glucose and lipid metabolism disorder and IR. After 4 weeks of pectin intervention, the FBG level of the db/db mice decreased significantly (Figure 1C), and the GSP level also decreased significantly. The change in GSP level could reflect the average blood glucose level of the body in the past 4–6 weeks, which is a key indicator to measure whether blood glucose is controlled or not, indicating that pectin has a certain effect on blood glucose control. In summary, pectin could reduce blood glucose levels by regulating the serum GSP level of db/db mice.

Beyond the observable manifestations of disrupted glucose metabolism, the inception of T2DM ushers in a concurrent perturbation in blood lipid regulation, lending it the fitting epithet of a “glyco-lipid disease” [33]. It is universally recognized that the functional and structural transformations in adipocytes bear a profound connection to the manifestation of IR. Metabolic disorders aggravate IR symptoms, usually manifested as abnormal increases in TC, TG, and LDL-C levels and abnormal decreases in HDL-C levels. In the present study, pectin significantly reduced the generation of TC and TG in the serum of the db/db mice and increased the level of HDL-C, thereby improving the blood lipid disorder of the db/db mice. Moreover, the adipocytes in the db/db mice were hypertrophic, and after gavage with pectin for 4 weeks, the volume of adipocytes became smaller, but there was little difference between the different groups. This indicated that pectin could improve lipid metabolism disorder in db/db mice. In addition to IR caused by abnormal blood lipid levels, it could also cause functional damage to islet cells [6]. Abnormal changes in blood lipids of the body would cause lipid toxicity in islet cells, increasing their apoptosis rate and ultimately destroying the normal life activities of islet cells. These results show that the number of islet cells in the pancreas of the MC group was small, and the structure and function of the cells were destroyed (Figure 4). After the intragastric administration of pectin, the number of islet cells in the pancreatic tissue increased, and the functions were restored. These results indicated that pectin could repair the functional damage of pancreatic tissue by improving blood lipid metabolism disorder and fat cell accumulation in db/db mice.

Numerous prior investigations have firmly established a compelling correlation between disruptions in the gut microbiota and the onset of metabolic syndrome, encompassing conditions such as obesity, type 2 diabetes, and non-alcoholic fatty liver disease [34,35]. Within this intricate interplay, natural plant polysaccharides emerge as significant influencers, wielding a noteworthy capacity to fine-tune the composition of the gut microbiota. The findings of the present study notably underscore that various pectin sources, when administered via gavage, have the remarkable ability to heighten the richness and diversity of the intestinal microbiota, along with effectively enhancing the *Firmicutes*/*Bacteroidetes* (F/B) ratio. This observed transformation in the gut milieu, with post-oral pectin administration, substantially translates into the augmentation of specific microbial players. Particularly, the relative abundances of *Ligilactobacillus*, *Lactobacillus*, and *Limosilactobacillus* experienced significant upswings within the PB and CP groups. Remarkably, dietary polysaccharides harnessed by the gut microbiota undergo fermentation and metabolic conversion, culminating in the generation of short-chain fatty acids (SCFAs) [18]. This metabolic phenomenon, in turn, serves as a catalyst for the proliferation of beneficial gut microorganisms, such as *Lactobacillus*. The observed result manifests as an appreciable increase in the cumulative content of SCFAs within the fecal matter of the experimental mice, distinctly exemplifying the capacity of orally administered pectin to induce this favorable shift within the gut ecosystem.

In general, pectin emerges as a potent agent in rectifying the disarray that characterizes blood lipid metabolism and the buildup of fat cells within db/db mice. This ameliorative action is chiefly achieved through the orchestration of multifaceted mechanisms. Pectin plays a pivotal role in calibrating the serum levels of GSP within db/db mice, thereby initiating a process of rehabilitation concerning pancreatic tissue function. Moreover, its influence extends to gut microbiota composition, inducing a favorable shift, and bolstering the concentration of SCFAs. This comprehensive approach culminates in the amelioration of both glucose and lipid metabolism disturbances in db/db mice, ushering in a more harmonious metabolic milieu.

## 5. Conclusions

The present study shows that pectin from *Citrus unshiu* Marc. decreased the levels of FBG, GSP, TG, TC, and LDL-C, whereas it increased the level of HDL-C in db/db mice, indicating that pectin could regulate glucose and lipid metabolism effectively. Pathological sections showed that the number of pancreatic islet cells and colonic goblet cells was increased in the db/db mice supplemented with pectin, and the volume of adipocytes was decreased. In particular, supplementation with pectin increased the ratio of F/B and the abundance of *Ligilactobacillus*, *Lactobacillus*, and *Limosilactobacillus*. Furthermore, pectin increased the content of acetic acid, propionic acid, and butyric acid in the gut of the mice. This comprehensive array of findings lends credence to the idea that pectin’s modulatory effects on gut microbiota composition and SCFAs serve as pivotal mechanisms in the enhancement of glucose and lipid metabolism. Collectively, these outcomes posit pectin as a promising prebiotic candidate for the prevention of disorders linked to glycolipid metabolism disturbances. While this study has unraveled these promising findings, future validation experiments will be pivotal in substantiating this phenomenon.

## Data Availability

Data are contained within the article.
